# Comparing *de novo *assemblers for 454 transcriptome data

**DOI:** 10.1186/1471-2164-11-571

**Published:** 2010-10-16

**Authors:** Sujai Kumar, Mark L Blaxter

**Affiliations:** 1 Institute of Evolutionary Biology, University of Edinburgh, West Mains Road, Edinburgh EH9 3JT, UK

## Abstract

**Background:**

Roche 454 pyrosequencing has become a method of choice for generating transcriptome data from non-model organisms. Once the tens to hundreds of thousands of short (250-450 base) reads have been produced, it is important to correctly assemble these to estimate the sequence of all the transcripts. Most transcriptome assembly projects use only one program for assembling 454 pyrosequencing reads, but there is no evidence that the programs used to date are optimal. We have carried out a systematic comparison of five assemblers (CAP3, MIRA, Newbler, SeqMan and CLC) to establish best practices for transcriptome assemblies, using a new dataset from the parasitic nematode *Litomosoides sigmodontis*.

**Results:**

Although no single assembler performed best on all our criteria, Newbler 2.5 gave longer contigs, better alignments to some reference sequences, and was fast and easy to use. SeqMan assemblies performed best on the criterion of recapitulating known transcripts, and had more novel sequence than the other assemblers, but generated an excess of small, redundant contigs. The remaining assemblers all performed almost as well, with the exception of Newbler 2.3 (the version currently used by most assembly projects), which generated assemblies that had significantly lower total length. As different assemblers use different underlying algorithms to generate contigs, we also explored merging of assemblies and found that the merged datasets not only aligned better to reference sequences than individual assemblies, but were also more consistent in the number and size of contigs.

**Conclusions:**

Transcriptome assemblies are smaller than genome assemblies and thus should be more computationally tractable, but are often harder because individual contigs can have highly variable read coverage. Comparing single assemblers, Newbler 2.5 performed best on our trial data set, but other assemblers were closely comparable. Combining differently optimal assemblies from different programs however gave a more credible final product, and this strategy is recommended.

## Background

Transcriptome sequencing projects for non-model organisms are popular because they cost less and are more computationally tractable than full genome sequencing projects, but still yield sufficient information to meet the requirements of many research programs. Traditionally, transcriptome projects have been based on Sanger dideoxy-sequenced expressed sequence tags (ESTs), but, because second-generation sequencing technologies provide much higher throughput than Sanger sequencing at a lower cost per base, these new technologies are increasingly used. For model organisms where a wealth of genomic information is available, the massively parallel, short-read (35-100 base) sequencing technologies (Illumina SOLEXA and ABI SOLiD) are most frequently used, as transcriptome reads can be mapped to the reference genome or transcriptome. However, most published non-model organism projects have used the Roche 454 pyrosequencing platform [1-36], because the longer reads generated (currently about 400 bases) are more amenable to *de novo *assembly and annotation.

A transcriptome project progresses through phases of data acquisition, assembly of the sequence reads to define putative transcripts, and then annotation and exploitation of the assembled data. The transcriptome assembly problem is not simple. Individual reads can have errors and polymorphisms that complicate recognition of overlaps, and individual transcripts (in non-normalised data) can have several orders of magnitude variation in abundance, and thus in effective coverage. Previous analyses of transcriptome data generated by Roche 454 pyrosequencing have almost always used just one software program for assembly (Table 1). Only two studies report the results of more than assembler. The first [1] briefly compared the length of assembled consensuses from Newbler [37], CAP3 [38], and stackPACK [39] on an *Arabidopsis thaliana *transcriptome dataset, whereas the second [11] demonstrated the comprehensive est2assembly pipeline on a phylogenetically diverse sample of insects, but only compared results for Newbler and MIRA [40]. Neither study provides a systematic comparison of assemblers, and so here we present such a comparison to assist the growing number of transcriptome projects using Roche 454 pyrosequencing data.

**Table 1:** Assemblers previously used for *de novo *assembly of 454 pyrosequencing transcriptome projects

Assembler	Organism
Newbler	*Arabidopsis thaliana *[1,2]; *Eucalyptus grandis *[3]; *Castanea dentata *and *Castanea mollissima *[4]; *Sarcophaga crassipalpis *[5]; *Acropora millepora *[6]; *Palomero toluqueño *[7]; *Eschscholzia californica *and *Persea americana *[2]; *Vitis vinifera *[8]; *Rhagoletis pomonella *[9]; *Heliconius spp*. [10]; *Euphydryas aurinia*, *Manduca sexta*, *Chrysomela tremulae*, *Papilio dardanus*, *Heliconius melpomene*, *Heliconius erato*, and *Melitaea cinxia *[11]; *Panax quinquefolius *[12]; *Sclerotium rolfsii *[13]; *Laternula elliptica *[14]
CAP3	*Zea mays *[15,16]; *Arabidopsis thaliana *[1]; *Ambystoma mexicanum *[17]; Human breast cancer [18]; *Artemisia annua *[19]; *Solanum arcanum *[20]; *Epimedium sagittatum *[21]; *Haemonchus contortus *[22]; *Laodelphax striatellus *[23]; *Coleochaete orbicularis *and *Spirogyra pratensis *[24]; *Bugula neritina *[25];
MIRA	*Centaurea solstitialis *[26]; *Chrysomela tremulae *[27,11]; *Pandinus imperator *[28]; *Zygaena filipendulae *[29]; *Manduca sexta *[30,11]; *Euphydryas aurinia*, *Papilio dardanus*, *Heliconius melpomene*, *Heliconius erato*, and *Melitaea cinxia *[11];
TGICL	*Pythium ultimum *[31]; *Zoarces viviparous *[32]; *Medicago truncatula *[33]
SeqMan	*Melitaea cinxia *[34]; *Cochliomyia hominivorax *[35]; *Pinus contorta *[36]
stackPACK	*Arabidopsis thaliana *[1]

Here we compare the performance of five assemblers: Newbler, CAP3, MIRA, SeqMan [41], and CLC's Assembly Cell [42] (Table 2). These assemblers differ in the algorithms used (most use variations of the Overlap-Layout-Consensus (OLC) strategy, while CLC uses de Bruijn graph path finding) and how they treat individual reads (whether a read is indivisible, or can be split and ultimately be placed in different contigs). We tested two versions of Newbler because we found the frequently-used, public release version (Newbler Version 2.3, hereafter referred to as Newbler 2.3) to have several undesirable features (see below) and thus contacted the developers to discuss these. They provided a pre-release version (Newbler Version 2.5 p1, hereafter referred to as Newbler 2.5) that addresses some of the issues identified. We did not include TGICL [43] or stackPACK [39] because both are essentially wrappers for the CAP3 assembler. Although Velvet [44] and ABySS [45] are popular assemblers for second-generation sequence data and can use Roche 454 pyrosequencing reads, they are primarily assemblers for genome sequence data from short-read platforms that rely on the high and even coverage depths afforded by these massively parallel technologies. In preliminary experiments on our data we were only able to generate very short contigs using short-read assemblers and so we have not compared Velvet, Oases (the transcriptome-specific version of Velvet, [46]), and ABySS.

**Table 2:** Features of assembly programmes compared in this study

Assembler	Type^†^	Splits reads*	Author	Cost	Source available	URL
CAP3	OLC†	No	X Huang and A Madan [38]	Free for use at non-profit organizations	No	http://seq.cs.iastate.edu/
CLC Assembly Cell 3.0	de Bruijn graph	Yes	CLC	Request quote or trial license	No	http://www.clcbio.com/
MIRA 3.0	OLC	No	Bastien Chevreux [40]	Free	Yes, GPL	http://sourceforge.net/projects/mira-assembler/
Newbler 2.3 and Newbler 2.5	OLC	Yes	Roche 454 [37]	Free for academic use	No	http://454.com/products-solutions/analysis-tools/gs-de-novo-assembler.asp
SeqMan NGen 2.1	OLC	No	DNAStar [41]	Request quote or trial license	No	http://www.dnastar.com/t-products-seqman-ngen.aspx

* i.e. data from one read can appear in multiple contigs

† OLC: Overlap-Layout-Consensus

Our target organism, *Litomosoides sigmodontis*, is a model filarial nematode, closely related to the causative agents of human filariases (*Brugia malayi*, *Wuchereria bancrofti *and *Onchocerca volvulus*). It originally derives from cotton rat hosts, but can be maintained in laboratory rodents (mice and gerbils) and is thus a tractable experimental system in which to investigate the dynamics of immune response induction and modulation, and test vaccine and drug candidates [47]. We expect *L. sigmodontis *to have a transcriptome similar to that of the filarial nematode *B. malayi *and the more distantly related rhabditid nematode *Caenorhabditis elegans*, with ~18,000 to 21,000 protein-coding genes generating ~30,000 different transcripts with mean length ~1.2 kb. The transcriptome project is part of a larger investigation into *L. sigmodontis *genomics, and detailed analyses of the content and biology of the transcriptome data will be published elsewhere.

## Results

### *L. sigmodontis *transcriptome data

We used a Roche 454 FLX instrument to generate 'standard chemistry' (mean read length ~220 bases) and 'Titanium chemistry' (mean read length ~350 bases) reads from cDNA libraries from three different lifecycle stages of *L. sigmodontis*: microfilaria (equivalent to the first stage larva of *C. elegans *and other nematodes), adult males, and adult females (Table 3, see Methods for more details on library preparation, read pre-processing, and assembly parameters). Reads were trimmed for adapters leaving a total of 741,387 reads with 205,065,666 bases used in all assembly experiments (trimmed read length histograms in Additional file 1, Figure S1).

**Table 3:** The *Litomosoides sigmodontis *transcriptome dataset read statistics

*L. sigmodontis *lifecycle stage	Technology	Number of reads	Number of raw bases	Number of trimmed reads	Number of trimmed bases	Mean length of trimmed reads	Median length of trimmed reads
Microfilaria (first stage larvae)	Titanium	366,813	203,227,223	351,387	118,039,337	335.92	374
Adult female	Standard	180,271	48,434,306	176,454	38,352,888	217.35	236
Adult male	Standard	216,940	59,231,575	213,546	48,673,441	227.93	245

**Total**	**Titanium + Standard**	**764,024**	**310,893,104**	**741,387**	**205,065,666**	**276.60**	**257**

### Comparison of assemblers

For each assembler, we used the default parameters recommended for transcriptome assembly (details are given in Methods). After assembly, contigs less than 100 bases in length and singletons were discarded for subsequent analyses. We compared the assemblies using the following standard metrics: total number of reads used in the assembly, number of contigs > 100 bases generated, N50 length of contigs (the smallest contig size in which half the assembly is represented), maximum contig length, summed contig length, and approximate time taken to perform analysis (Table 4). We include the N50 as a measure even though it is not strictly appropriate for transcriptome assemblies (where we expect the median contig length to be in the region of 1.2 kb). We also assessed assembly integrity and completeness by comparison to four reference datasets.

**Table 4:** Basic assembly metrics

	CAP3	CLC	MIRA	Newbler 2.3	Newbler 2.5	SeqMan
Number of contigs†	24727	22746	35827	12019	21734	29969
Total Bases	16733217	14875522	21339704	14456476	20066883	21355682
Number of contigs (> = 1 kbp)	4403	4174	4770	6320	7661	6082
Total Bases (in contigs > = 1 kbp)	6461079	6255785	7027775	10810962	13691429	9296011
Max contig length	4011	4368	5784	5872	6228	6263
Mean contig length	677	654	596	1203	923	713
N50	806	850	708	1487	1448	880
Number of contigs in N50	6533	5459	9148	3406	4649	7555
Reads used (SSAHA2)	670425	679152	672036	616672	667597	681974
Multi-hit reads (SSAHA2)	271648	118334	392884	249210	352887	322409
Reads used (CLC)	690889	691818	696527	600132	681831	711726
Multi-hit reads (CLC)	91951	24485	162365	213670	262178	128631
Time taken	1 day*	4 minutes *	3 days *	2 hours *	45 minutes *	6 hours **

† only contigs > 100 bases were assessed

* on a dual quad-core 3 GHz Xeon workstation with 32 GB RAM

** on a dual core 2.53 GHz Mac mini server with 4 GB RAM

### Numbers of reads used in assembly

The optimal assembler will use all the reads given, and will deliver assemblies with unambiguous mappings of reads to contigs, and thus putative transcripts. Each assembler has a different way of reporting the number of reads utilised. For example, MIRA reported only 2,170 singletons but classified 115,688 unassembled reads as 'debris'. Similarly, Newbler 2.3 and Newbler 2.5 generate separate lists of singletons, repetitive reads, and 'outliers' (problematic reads such as chimaeras). CAP3 and SeqMan only report assembled and unassembled reads. CLC does not track reads at all and maps reads back to the assembly to estimate where they might belong. Therefore, to compare the assemblies, we mapped all the reads back to each assembly using SSAHA2 [48] and the CLC reference aligner (which is part of the Assembly Cell suite [42]), as recommended in a study comparing short-read aligners [49]. SSAHA2's default settings for Roche 454 reads are clearly tuned for sensitivity, because the number of reads with multiple matches is very high compared to the CLC aligner. For both aligners, the highest numbers of reads were mapped back to the SeqMan assembly, while fewest were mapped to the Newbler 2.3 assembly. CAP3, CLC, and MIRA were comparable in terms of the number of reads used. CLC had the fewest reads with multiple matches by far, indicating that it was the least redundant of the six assemblies.

### Number, mean length, and summed length of contigs

**Figure 1:**
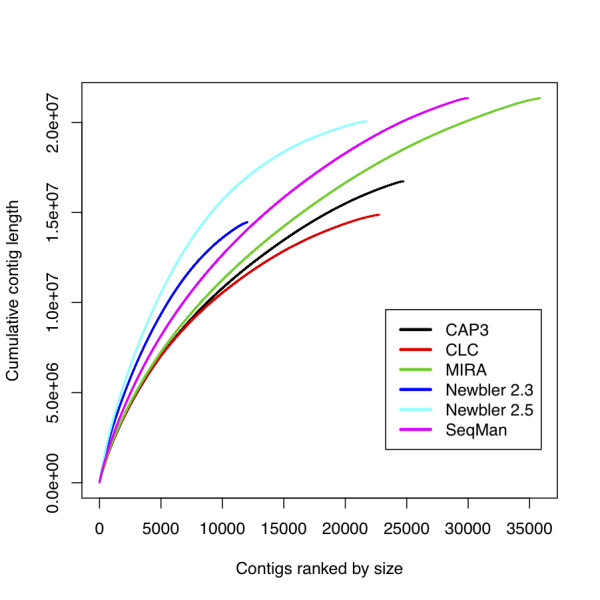
**Cumulative contig lengths generated by different assembly programs**. For each of six assemblies, contigs longer than 100 bases were ordered by length, and the cumulative length of all contigs shorter than or equal to a given contig was plotted. The total length of the assembly and the number of contigs present in the assembly define the end point of each curve, while the initial slope of each curve reflects the proportion of longer contigs.

The optimal assembler will produce the longest summed length of contigs, while avoiding over-assembly of reads into *in silico *chimaeras, and avoiding the production of near-identical, largely overlapping contigs from allelic copies or error-rich data. It will also produce a transcriptome estimate with a mean and variance in contig length similar to that expected from the whole transcriptome. Newbler 2.3 generated an assembly with the largest N50 and the longest mean contig length (Table 4), but it also produced the smallest assembly span of the six programmes tested. Inspection of contig assemblies using the next-generation sequence assembly visualisation software Tablet [50] showed that Newbler 2.3 was discarding read overlap information in deriving the assembled contig sequence. The overall assembly sizes of CAP3, CLC and Newbler 2.3 are comparable to each other (between 14.4 and 16.7 Mb), as are the assembly sizes of MIRA, Newbler 2.5 and SeqMan (between 20.0 and 21.3 Mb). Newbler 2.5 has an assembly that is 39% larger than the Newbler 2.3 assembly, and seems to have solved the previous version's problem of discarding sequence data. Of all the six assemblers, Newbler 2.5 produced the highest number of contigs longer than 1 kb, and the most bases in contigs longer than 1 kb (these larger overall contig sizes are represented in Figure 1 as the assembly with the steepest initial slope). MIRA and SeqMan both generated comparable assembly spans, but with at least 8,000 more contigs than Newbler 2.5, indicating that they have shorter contigs overall.

### Speed of analyses

The optimal assembler will complete analyses in a short period of time, and the time taken will scale well with increasing data volumes. While speed itself is not an overriding optimality criterion, rapid analyses will permit robust and extensive exploration of parameter space. In addition, some algorithms can make efficient use of multi-threaded processors or cluster computing, effectively reducing their wall-clock run time. The speed metric is not linked to assembly quality. CLC was astonishingly fast (taking only a few minutes to assemble 741,387 reads) compared to MIRA (which took 3 days in 'accurate' mode on the same hardware). This speed comes at a significant cost however, as CLC does not track read placement in the assembly. Tracking of read placement in an assembly is a very valuable feature, as it allows inspection of the data underpinning suspect assemblies, and thus CLC sacrifices speed for verifiability.

### Assembly redundancy, shared and novel bases

**Figure 2:**
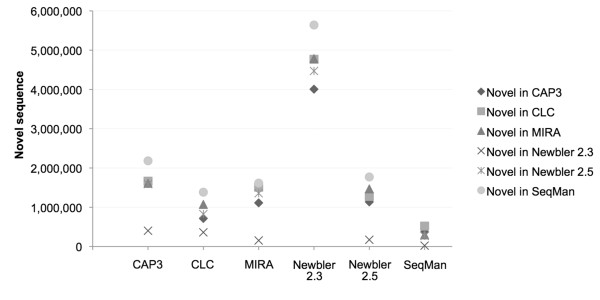
**Novel sequence in pair-wise comparisons between assemblies produced by different assemblers**. For each assembly, we calculated the number of bases in the other assemblies that were not present in the focal assembly.

In comparison to other assemblies, the optimal assembly will include the largest proportion of the unique bases present in the sum of all assemblies. Thus an assembler that produced a large assembly that included many contigs that were redundant could be worse that an assembly that was shorter but only included unique bases. To determine if the differing assembly sizes were due to novel sequences in each assembly, or just due to repetitive and redundant assemblies, we used BLAT [51] with default parameters to pairwise align all six assemblies (Figure 2).

These pairwise comparisons show that the SeqMan assembly had the most novel bases when compared to the others, and the others five assemblies had the fewest novel bases when compared to SeqMan. At the other end of the spectrum, the Newbler 2.3 assembly had the fewest novel bases and almost all its bases were seen in the other assemblies. For example, the SeqMan assembly had over 5 Mb of novel sequence compared to the assembly from Newbler 2.3, and the latter had only 23,366 novel bases in the reciprocal comparison. CAP3, CLC, MIRA, and Newbler 2.5 were all very similar in that they generated assemblies that had about 1-2 Mb of novel sequence compared to each other.

SeqMan, MIRA and Newbler 2.5 all generated assemblies in excess of 20 Mb, 25-50% larger than the 14-16 Mb assemblies produced by CAP3, CLC, and Newbler 2.3. However, these additional contigs and bases did not represent an excess of novel sequence, except in the case of SeqMan, suggesting that MIRA and Newbler 2.5 produce some redundant contigs (identical except for real polymorphisms or sequencing error). CLC generated the least redundant assembly, a direct consequence of using a de Bruijn graph algorithm with small fragments (21-mers) that collapses most repeats.

### Alignments to reference sequences

Another optimality criterion for a novel *de novo *assembled transcriptome is how well it recapitulates previously determined sequences for the target species, and how well it represents sequences from related organisms. The best assembler will return contigs that match previous data well, and will deliver a high coverage of the conserved proteome of related taxa. We used four comparator datasets:

(i) a set of 2699 Sanger dideoxy sequenced ESTs from *L. sigmodontis *(Mark Blaxter, unpublished), assembled into 1602 clusters spanning 0.9 Mb (see http://www.nematodes.org/nembase4/species_info.php?species=LSC). We expect the Roche 454 pyrosequencing assemblies to faithfully reflect these longer reads and clusters;

(ii) the proteome predicted from the genome sequence of *B. malayi *[52]. We expect the *B. malayi *RefSeq protiens (11,472 GenBank entries, totalling 4.3 M amino acid residues) to be a good model for the proteome of *L. sigmodontis*, and thus proportional coverage of this proteome should reflect assembly quality;

(iii) gene families conserved across the phylum Nematoda derived from MCL-TRIBE [53] analysis of 62 species assembled in NEMBASE4 database (Mark Blaxter and Ben Elsworth, unpublished; see http://www.nematodes.org/nembase4/tribe_tree.php). We selected 3,681 tribes, containing 120,926 EST clusters, that had representatives in species spanning the inferred base of the phylum. They represent an estimate of the conserved core of genes that may be present in all nematode proteomes, and again proportional coverage should reflect assembly quality.

(iv) a set of 9,782 *C. elegans *proteins, grouped into 3,731 euKaryotic Ortholog Groups (KOGs), that are known to have orthologs in the proteomes of six other eukaryotic genomes [54]. We expect mRNAs for these conserved proteins to be present in the *L. sigmodontis *data, and thus proportional coverage should reflect assembly quality.

These datasets are not independent, but each offers a different assessment of assembly quality. Tables 5, 6, 7 and 8 show the results of aligning the contigs from each assembly to each of these four datasets. In all four cases, the best assembler was either Newbler 2.5 or SeqMan. Newbler 2.3 had the lowest number of significant matches to known reference and related sequences. However, to identify which assembler had the largest number of well-assembled long contigs, we did a second assessment of each dataset where only those hits that covered at least 80% of a target sequence were considered. With this restriction, CLC had the fewest hits in all cases except hits to conserved nematode proteins (Table 7), suggesting that the longer contigs from the CLC assembly were not as accurate.

**Table 5:** BLAT hits to 1602 *Litomosoides sigmodontis *EST clusters

	CAP3	CLC	MIRA	Newbler 2.3	Newbler 2.5	SeqMan
**% of ESTs hit by BLAT**	87.8	90.3	89.6	81.6*	89.6	90.8
**(% of bases covered)**	(78.2)	(80.0)	(80.1)	(71.3)*	(78.7)	(82.0)
**% of ESTs hit by BLAT where each hit covered at least 80% of the target EST sequence**	59.9	51.9	62.5	59.4	63.9	65.4
**(% of bases covered)**	(59.1)	(50.5)*	(61.1)	(59.4)	(63.7)	(64.3)

* indicates a value significantly lower than the others, using a Huber M-estimator.

**Table 6:** BLASTX hits to 11,472 *Brugia malayi *proteins

	CAP3	CLC	MIRA	Newbler 2.3	Newbler 2.5	SeqMan
**% of proteins hit**	76.7	78.4	77.3	68.9*	77.9	78.6
**(% of bases covered)**	(60.4)	(62.4)	(59.7)	(51.8)*	(61.5)	(63.0)
**% of protein hit by individual HSPs that cover 80% of target protein**	27.0	26.0	26.5	29.2	32.4	28.7
**(% of bases covered)**	(16.8)	(16.1)	(16.4)	(19.9)	(22.3)	(18.0)

Note: E-value cutoff 1e-5

* indicates a value significantly lower than the others (p < 0.01), using a Huber M-estimator.

**Table 7:** BLASTX hits to 3,681 tribes containing 120,926 conserved nematode proteins

	CAP3	CLC	MIRA	Newbler 2.3	Newbler 2.5	SeqMan
**% of unique tribes hit**	91.7	92.2	91.4	87.0*	92.0	92.4
**% of unique tribes hit where individual HSPs covered 80% of target protein**	81.7	81.1	78.9	77.1*	82.1	81.6

Note: E-value cutoff 1e-5

* indicates a value significantly lower than the others (p < 0.01), using a Huber M-estimator.

**Table 8:** BLASTX hits to 3,731 KOGs containing 9,782 *C. elegans *proteins

	CAP3	CLC	MIRA	Newbler 2.3	Newbler 2.5	SeqMan
**% of unique KOGs hit**	89.2	89.7	88.6	83.7*	89.7	90.1
**% of unique KOGs hit where individual HSPs covered 80% of target protein**	30.2	27.1	28.6	33.0	35.7	30.4

Note: E-value cutoff 1e-5

* indicates a value significantly lower than the others (p < 0.01), using a Huber M-estimator.

### Merging assemblies to improve credibility

As each assembler uses a different algorithm to derive final contigs, and each of these algorithms may model different portions of the true transcriptome with different accuracies, we reasoned that combining assemblies might identify the subset of the assemblies that had high credibility, as it was found by both (or multiple) approaches. We checked if pairs of assemblies performed better than individual assemblies under the various optimality criteria proposed above, particularly in their recapitulation of the reference data. We combined two assemblies at a time by treating their (first-order) contigs as pseudo-reads and assembled these using a traditional OLC assembler (CAP3 with default settings). Only second-order contigs that contained first-order contigs from both constituent assemblies were considered for further analysis because two independent assemblers had agreed on the consensus sequence in that contig: we term these "robust contigs".

Whereas previously the six assemblies had generated from 12,000 to 36,000 contigs and spanned 14.5 Mb to 21.4 Mb (Table 4), we found that merging assemblies resulted in a much narrower range of contig numbers and total span (Table 9). The N50 and mean contig sizes also increased, approaching that predicted for a complete transcriptome.

**Table 9:** Secondary assemblies by merging pairs of initial assemblies using CAP3 with default settings

Assembly 1	Assembly 2	Number of "Reads" (contigs) in Assembly 1	Bases in Assembly 1	Number of "Reads" (contigs) in Assembly 2	Bases in Assembly 2	Number of second-order contigs with "reads" from both assemblies	Bases in second-order contigs with "reads" from both assemblies
MIRA	SeqMan	35827	21339704	29969	21355682	18068	16293192
MIRA	Newbler 2.5	35827	21339704	21734	20066883	15951	15866051
Newbler 2.5	SeqMan	21734	20066883	29969	21355682	15783	15701053
CLC	Newbler 2.5	22746	14875522	21734	20066883	15778	15825663
CAP3	MIRA	24727	16733217	35827	21339704	15688	14243534
CLC	SeqMan	22746	14875522	29969	21355682	15504	14679975
CAP3	SeqMan	24727	16733217	29969	21355682	15387	14824287
CLC	MIRA	22746	14875522	35827	21339704	15334	14357031
CAP3	Newbler 2.5	24727	16733217	21734	20066883	14275	14830304
CAP3	CLC	24727	16733217	22746	14875522	14149	13753398
Newbler 2.3	Newbler 2.5	12019	14456476	21734	20066883	9733	13252303
MIRA	Newbler 2.3	35827	21339704	12019	14456476	9380	11731374
CLC	Newbler 2.3	22746	14875522	12019	14456476	8884	12318589
CAP3	Newbler 2.3	24727	16733217	12019	14456476	8484	11426423
Newbler 2.3	SeqMan	12019	14456476	29969	21355682	8274	11452990

These robust contigs also performed better in the tests assessing representation of reference datasets (Table 10 and Table 11). This was especially true for matches filtered to include only those covering at least 80% of the reference sequence. The co-assemblies of [CLC and MIRA] and [MIRA and Newbler 2.5] matched the most EST clusters. The [CLC and Newbler 2.5] combined assembly matched the most *B. malayi *peptides.

**Table 10:** Alignments to 1602 EST clusters where > 80% of the EST was covered by a match, by pairs of assemblies merged using an OLC assembler

Assembly pair		% EST clusters hit	% EST bases covered
CLC	MIRA	65.4	65.1
MIRA	Newbler 2.5	65.4	65.3
MIRA	SeqMan	64.9	64.6
CLC	Newbler 2.5	64.7	64.6
Newbler 2.5	SeqMan	63.2	62.9
CAP3	Newbler 2.5	63.0	62.6
CAP3	CLC	62.9	62.4
CLC	SeqMan	62.9	62.5
CLC	Newbler 2.3	62.4	62.5
CAP3	MIRA	62.2	61.9
CAP3	SeqMan	61.7	61.3
MIRA	Newbler 2.3	61.7	61.9
Newbler 2.3	Newbler 2.5	61.4	61.5
Newbler 2.3	SeqMan	60.0	60.2
CAP3	Newbler 2.3	59.7	59.6

**Table 11:** Alignments to 11,472 *B. malayi *peptides using BLASTX (e value < 1e-5) where > 80% of the protein was covered by a match, by pairs of assemblies merged using an OLC assembler

Assembly Pair		% Peptides hit	% Peptide bases covered
CLC	Newbler 2.5	38.2	28.3
MIRA	Newbler 2.5	37.3	27.4
CLC	MIRA	37.1	27.1
CLC	Newbler 2.3	36.5	27.4
Newbler 2.5	SeqMan	36.5	26.8
CAP3	Newbler 2.5	36.4	26.9
CAP3	CLC	36.1	26.3
CLC	SeqMan	35.9	26.1
MIRA	SeqMan	35.7	25.7
Newbler 2.3	Newbler 2.5	35.3	26.3
CAP3	SeqMan	35.1	25.2
Newbler 2.3	SeqMan	34.4	25.6
CAP3	MIRA	34.0	24.2
CAP3	Newbler 2.3	34.0	25.1
MIRA	Newbler 2.3	33.3	24.0

Although there was no consistent pattern indicating a clear superiority of any pair of assemblies (other than the exclusion of the Newbler 2.3 assembly), these merged assemblies show that it is possible to get more long, robust contigs that align better to reference sequences by combining the outputs of two different assemblies. Co-assembling three primary assemblies at a time and considering only the contigs that had 'reads' from all three assemblies gave smaller total contig numbers than pairwise co-assemblies. The number of stringent matches to reference sequences went up by less than 1% in all cases (see Additional File 2, Tables S2-S7), suggesting that the additional effort involved in generating and combining these assemblies may not be worthwhile.

### Summary

We compared six assemblers by aligning their assembly contigs to four reference sequence sets. The Newbler 2.3 assembly scored worst in each comparison, probably because it had the smallest span of all the assemblies. Under stricter comparison parameters, the CLC assembly was poorer, suggesting that this assembly is more fragmented than the others. Although no single assembler was optimal in every case, Newbler 2.5 and SeqMan had the best alignments to related reference sequences overall. However, SeqMan generated over 8000 contigs extra for approximately the same total number of assembled bases (21.4 Mb versus 20.1 Mb).

We were able to improve the assembly by merging two assemblies at a time using a traditional OLC assembler (CAP3). This approach generated more high quality alignments to our reference sets.

## Discussion and Conclusions

We compared six programmes (Table 2) for the task of *de novo *assembly of transcriptome data from an organism with little or no previous genomic resources. We tested each using default or minimally adjusted parameters, as has been the common practice in previous 454 pyrosequencing transcriptome studies (Table 1). It may be that it is possible to generate better assemblies for particular datasets by exploring the parameter space for each assembler. However, as sequencing becomes cheaper and more accessible, the vast majority of 454 transcriptome assemblies will probably be done by researchers who are new to assembly and just want something that works with a few sensible default parameters. Of the six *de novo *transcriptome assemblers tested, Newbler 2.5 had the best contig length metrics for our data, and, along with SeqMan, had the best alignments to related reference sequences.

Each assembler has certain advantages and disadvantages that are presented in more detail in the Supplementary Materials (Additional file 1). Versions 2.3 and 2.5 of Newbler were the only assemblers that explicitly attempted to reconstruct and group alternative transcripts and isoforms. If only one assembler had to be used because of time or resource constraints, we would currently recommend Newbler 2.5 overall. However, none of the other assemblers are deprecated apart from Newbler 2.3. We strongly recommend redoing transcriptome assemblies that were performed with Newbler 2.3 or earlier versions, if the goal is to get as complete an assembly as possible.

It is possible that different raw data sets may be better assembled by different programs (e.g. MIRA may be better for normalized transcriptome sequence data, and CAP3 for paired transcriptome data), and thus researchers should perhaps cross-compare the best available ones on their data using the optimality criteria used here.

Merging assemblies performed with different programs is a frequently used approach in genome assembly projects, especially those that employ multiple sequencing technologies. Application of this strategy to the problem of *de novo *transcriptome assembly appears particularly useful. While a merged assembly may simply sum the errors made by both programs, filtering the resultant second stage contigs on the basis of their containing first stage contigs from both of the starting sets generates a transcriptome assembly that is on average longer and that better represents known or assumed reference sequences than do either of the starting contig sets. A preferred assembly strategy is thus to perform initial assemblies with multiple high-quality assemblers and then to merge these using a traditional OLC assembler such as CAP3. Second-order contigs that have support from multiple first-order assemblies are much more likely to be accurate.

### Need for new assemblers

All the *de novo *assemblers in this study, with the exception of CLC, use the OLC assembly strategy. CLC uses de Bruijn graphs. As read lengths and throughput increase and sequencing costs come down, an average transcriptome assembly project for a non-model organism may comprise several million 800 base reads. Under the OLC paradigm, the computational time for assembling more reads rises exponentially with data complexity because the number of pairwise comparisons to detect overlaps will increase, and the layout graphs will be harder to resolve into a consensus sequence. The CLC *de novo *assembler is clearly a step in the right direction because its de Bruijn graph algorithm achieves reasonable results in very little time on large datasets. Other de Bruijn graph assemblers such as Oases [46] and ABySS [55] are promising but are currently not suitable for most non-normalised 454 pyrosequencing *de novo *transcriptome assembly projects because many transcripts have very low coverage that cannot be assembled reliably. Perhaps the way forward is to use the de Bruijn graph method for transcripts with high coverage and the OLC method for transcripts with low coverage. We have shown that an assembly-merging strategy delivers robust contigs from intermediate assemblies produced by current programs, and this strategy is likely to be of utility in deriving the best assemblies from future programs as well.

## Methods

### Library preparation

The *L. sigmodontis *samples were prepared by Stella Konstantinou, Eleana Theophilou and Simon Babayan, and sequenced by the GenePool, Edinburgh. Briefly, total RNA from three *L. sigmodontis *samples (adult male, adult female, microfilaria) was converted to double stranded cDNA using Evrogen's MINT cDNA synthesis kit. First strand cDNA was synthesised using reverse transcriptase (RT) from a 3'-primer with oligo(dT) sequence that annealed to the poly-A stretch of RNA and synthesised cDNA until the 5' end of the mRNA. Finally, double stranded cDNA synthesis was performed using PCR amplification, and the final product contained the same MINT adapter sequence at both 3' and 5' ends. The cDNA was fragmented, size-selected, library-prepped, and sequenced according to standard Roche-454 FLX and Titanium protocols. Sequence files have been deposited into the Sequence Read Archive (SRA) with accession number ERA011678 http://www.ebi.ac.uk/ena/data/view/ERA011678.

### Read pre-processing

We chose to remove MINT adapters from the sequences ourselves rather than use the built-in adapter removal tools in MIRA, Newbler, SeqMan, and CLC, because each assembler pre-processes reads slightly differently and CAP3 does not remove adapters at all. The Roche 454 instrument outputs sequences in Standard Flowgram Format (SFF) files that come with quality and clipping information. To ensure that each assembler got exactly the same input, we changed the trim points directly in the SFF file to exclude adapter sequence rather than first convert the SFF files to fasta and quality files. The 29 base MINT adapter sequence was identified using BLASTN [56] from NCBI's new blast+ suite (Version 2.2.23) and by masking all matches with a bit-score greater than an empirically determined threshold of 25. The longest unmasked portion of the sequence was used to set new trim points in the SFF file.

Most EST pipelines remove poly(A/T) regions from reads because aligners would not be able to find good matches back to genomes that do not have the poly(A/T) sequence, and assemblers might try to align on these sequences. The former reason was not a factor for us as this was an unsequenced organism, but we did a trial run both with and without the poly(A/T) sequence and found no misassemblies on these regions by any of the assemblers. Newbler and MIRA account for such sequences when run in the -cdna and EST modes respectively. SeqMan and CAP3 remove low quality ends (homopolymer runs of poly(A/T) sequence have lower qualities in 454 sequencing), and CLC uses a de Bruijn graph which would not allow misassemblies because it would not be able to resolve the branching structure in a poly(A/T) region. Poly(A/T) sequence might also be indistinguishable from genomic sequences in some cases and therefore should not be removed. We found that the total amount of assembled sequence was about 10% lower for assemblies without poly(A/T) sequence. Therefore, we did not trim the poly(A/T) regions in the sequences. An additional advantage of leaving the poly(A/T) sequence in is that assembled contigs with a poly-A at the end (or poly-T at the start) indicate that the assembly end point is almost certainly correct. In our sequences, a poly(A/T) sequence typically had an N about half-way through because a degenerate oligo-dT primer was used to extract the poly-A tails from the cDNA. Any sequences with more than one N were discarded.

### Assembly parameters

For each assembler, the version and parameters used are described in this section. CLC, Newbler, and SeqMan used the trimmed SFF files directly. CAP3 and MIRA used fasta and quality files extracted from the SFF files using Roche 454's sffinfo utility. All the assemblers returned a fasta file with contigs, and, except CLC, they all returned an ACE file with read placement information for each contig. All assemblers were run on an 8-core 3.0 GHz Linux workstation with 32 GB of memory, with the exception of SeqMan NGen, which currently runs only on Mac and Windows operating systems. SeqMan NGen was run on a dual core 2.53 GHz Mac mini server with 4 GB of memory.

The CAP3 binary for 64-bit Intel Linux systems was downloaded on June 20, 2010 from http://seq.cs.iastate.edu/cap3.html. The version was not specified in this download, and we did not change any of the default settings.

The CLC Assembly Cell command line application (version 3.02) has only one assembly-specific parameter, the minimum contig length reported, which we set to 100. It converts Roche 454 .sff files to fasta format, strips out all read identifiers, performs the assembly, and returns only a fasta file with contigs. As it does not keep track of reads, we had to use the CLC aligner, also known as the Reference Assembly application, to perform the additional step of mapping all the input reads back to the assembly contigs using default settings.

MIRA version 3.0.0 (production version) was used with the recommended quick switches for a Roche 454 EST assembly: -job = denovo,est,accurate,454. We used the -notraceinfo option because the traceinfo file provides clipping information, whereas we had already used the clipping information to create trimmed fasta and quality files. We also used -GE:not = 8 which is a general option for specifying the number of threads that should be used for steps that can use multiple cores. MIRA's final contig output file includes singletons but we looked for and removed all contigs with the "_s" prefix ("_c" indicates contigs with more than one read).

The latest releases of Roche 454's Newbler (version 2.3, 091027_1459, and version 2.5, newbler v2.5p1-internal-10Jun23-1) provide a "-cdna" option to assemble transcriptomes. In transcriptome assembly, the assembler frequently fails without the -cdna option because it expects approximately even coverage in genome assembly mode. The only other parameters we used were "-ace" (to generate an ACE file at the end) and "-cpu 8" to use all available cores. Like most other OLC assemblers, Newbler stops extending contigs when it cannot resolve branches in the overlap layout graph. Unlike other assemblers, Newbler then tries to create isotigs out of contigs that are consistently connected by a subset of reads. Each isotig corresponds to an alternative transcript, and any contigs or isotigs that share any read overlaps are put into the same isogroup. We noticed that several contig fragments in the Newbler 2.3 ACE file were not reported in the final assembly fasta file (454Isotigs.fna). Initially, the Newbler developers said that these contigs are not parts of actual transcripts and are therefore not reported, but we traced a few of these fragments and found that they were made up of well aligned, overlapping reads that had been split into contigs and then discarded. The developers have since modified Newbler and allowed us to try the pre-release version on our data that addresses this issue, using the -urt flag that allows contigs to be bridged across regions with only single-depth coverage.

DNAStar's SeqMan Ngen version 2 for Mac has a GUI that calls an underlying script. We chose the *de novo *transcriptome assembly option for 454 unpaired reads. When we tried a run with default settings and no quality or vector trimming, the assembly completed without any error messages but reported only 891 contigs. Subsequently we tried an assembly with quality trimming enabled which completed in 6 hours and reported a 21 Mb assembly.

### Assembly read alignment

To get assembly stats on the number of reads used, we used SSAHA2 [48] and CLC's aligner to map all the input reads back to each assembly's contigs (as recommended in a comparison of next-generation aligners [49]). SSAHA2 was used with the -454 option, and the clc_ref_assemble_long binary from CLC's Assembly Cell distribution version 3.00.44070-beta2 was used with default settings.

## Abbreviations

OLC: Overlap Layout Consensus; EST: Expressed Sequence Tag; kb: kilo basepairs; Mb: mega basepairs; MB: mega bytes; GB: giga bytes; KOG: euKaryotic Orthologous Group; HSP: High Scoring Pair, a query sequence can have only one hit to a target sequence in a blast database, but each hit can be made up of many HSPs

## Competing interests

The authors declare that they have no competing interests. Although we have used commercial software in this study supplied by the manufacturers, they had no influence on the experiments performed nor on the results reported here.

## Authors' contributions

SK and MB conceived and designed the project. Analyses were carried out by SK and reviewed by MB. SK and MB wrote the manuscript. All authors read and approved the final manuscript.

## Supplementary Material

Additional file 1 **Supplementary Materials**. Figure S1: Trimmed read length histograms. A section describing the advantages and disadvantages of each assembler. Click here for file

Additional file 2 **Results of merging three assemblies**. Assembly metrics as a result of merging three first-order assemblies. Alignments of merged assemblies to reference sequences. Click here for file
