# Supplementary material for: Comparing de novo assemblers for 454 transcriptome data
Source: BMC Genomics. 2010 Oct 16;11:571. doi: 10.1186/1471-2164-11-571 (PMC3091720; doi:10.1186/1471-2164-11-571)
Supplement: Additional file 1 — Supplementary Materials. Figure S1: Trimmed read length histograms. A section describing the advantages and disadvantages of each assembler. [file 1471-2164-11-571-S1.DOC]

**Supplementary Materials**

A
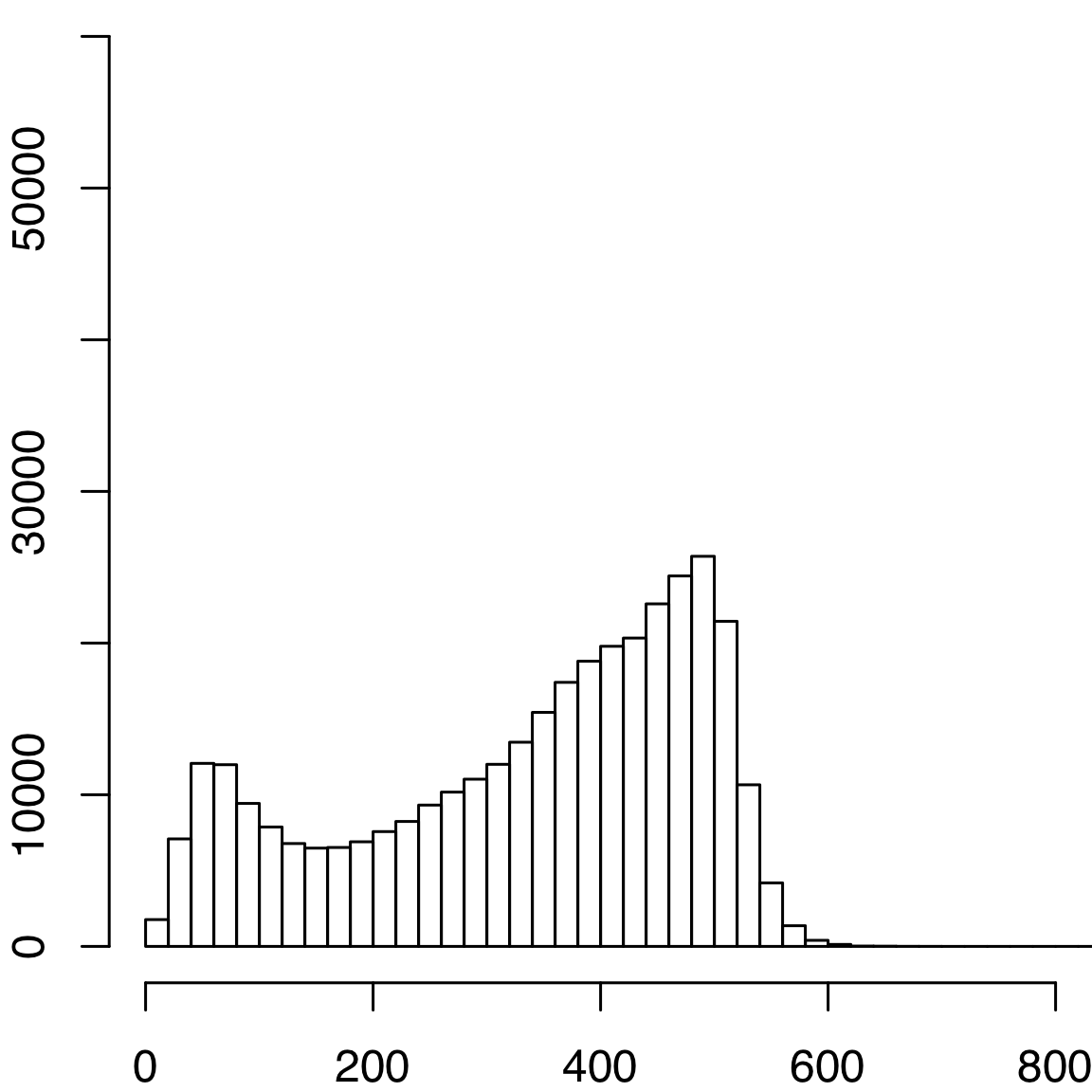
 B
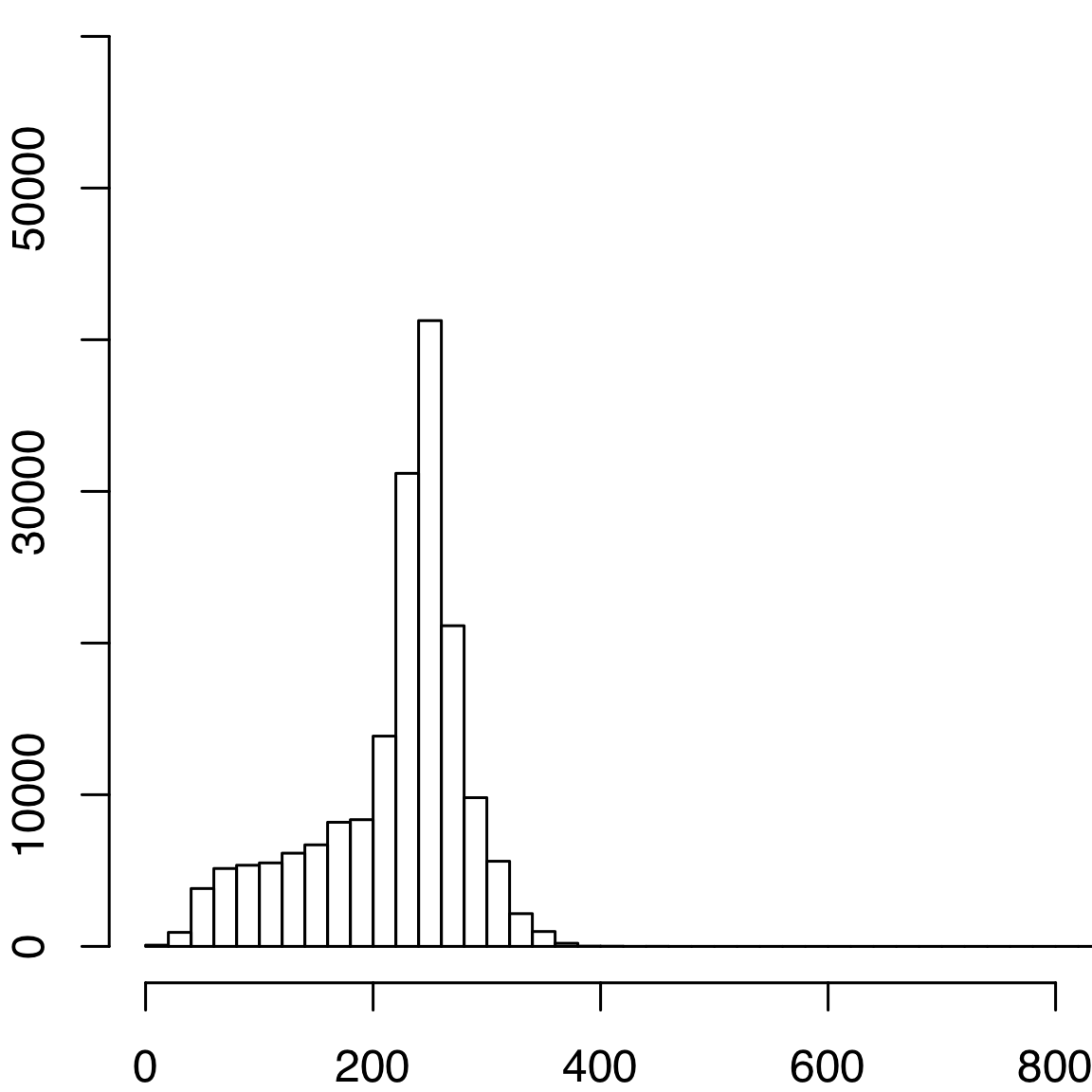


C
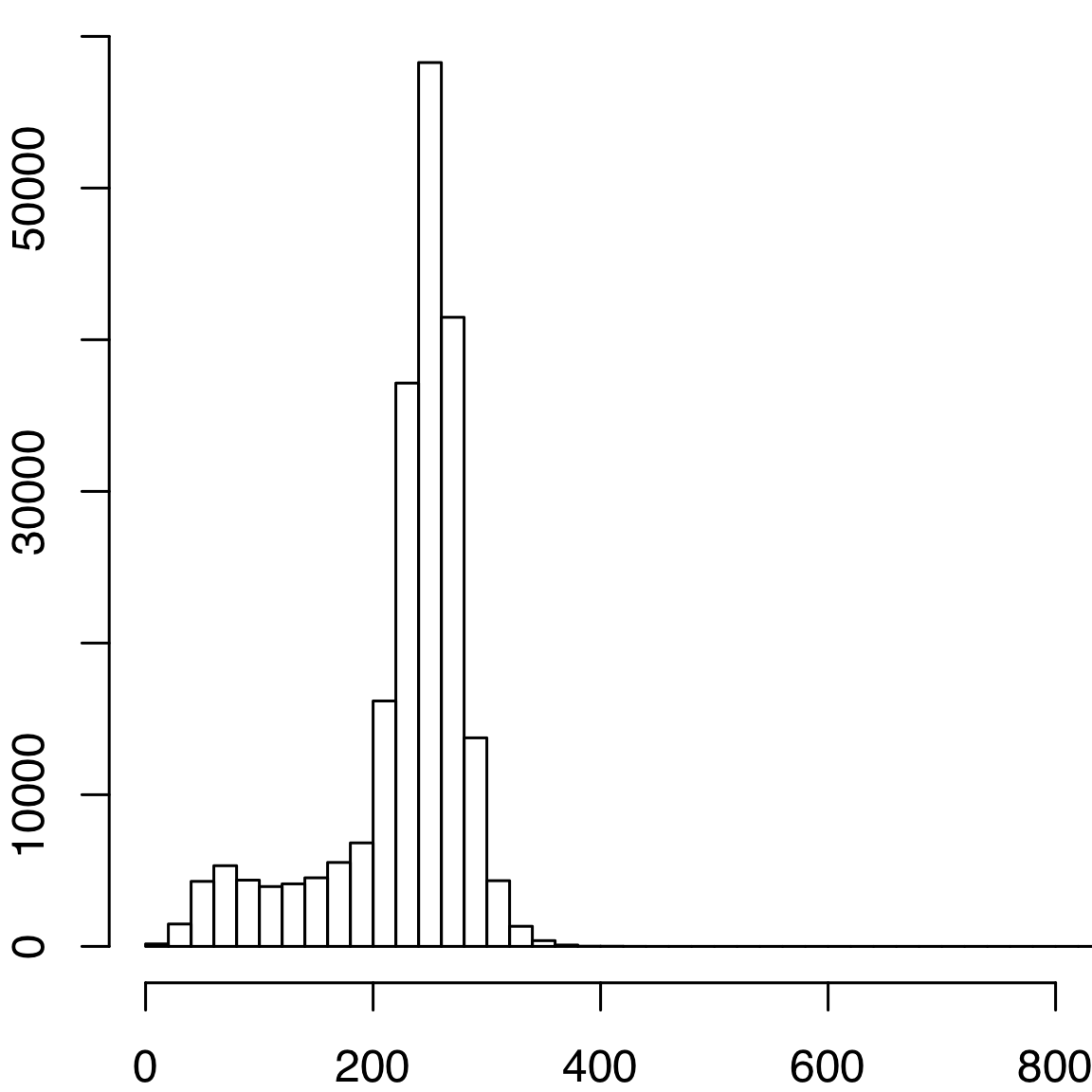


**Figure S1.** Read length histograms for trimmed reads from three *Litomosoides sigmodontis* transcriptome libraries: (A) Microfilaria (first stage larvae), 454 Titanium chemistry; (B) Adult female, 454 FLX standard chemistry; (C) Adult male, 454 FLX standard chemistry.

**Advantages and disadvantages of each assembler**

In terms of features and flexibility, MIRA is the most comprehensive assembler with the highest number of user-definable parameters, but it is slow and, as the manual admits, "sequencing errors sometimes interfere heavily when coverage exceeds 80x" [1], a situation often found in our non-normalised dataset. MIRA also had the highest redundancy. MIRA is recommended for analysis of a normalised data set or a filtered set of reads that did not have extreme coverage, but not for a representative dataset such as the *L. sigmodontis* one analysed here.

CLC is the fastest assembler by far and used the least amount of memory (350 MB at peak usage). The CLC Assembly Cell User Manual [2] states that it achieves this remarkable speedup over other assemblers by utilising de Bruijn graphs (also used by Velvet, ABySS and most new short-read assemblers) rather than the traditional OLC paradigm, by efficiently using multiple cores, and by optimising low level machine code (i.e. it uses assembly language for assembly). De Bruijn graph assemblers split reads into overlapping k-mers and utilize short reads with high coverage efficiently to make large contigs possible, but for transcriptome datasets where different contigs may have varying and often low coverage, they are unlikely to be optimal. CLC determines the k-mer automatically, but only up to a maximum of 31, which probably explains the fragmented assembly. Another drawback of the use of small k-mers is that they cannot recreate alternate transcripts, because any alternate path is broken at the branch points in the de Bruijn graph, and each fragment is reported as a separate contig. The CLC developer team also confirmed to us that their assembler keeps all k-mers with a coverage >1, a feature that probably explains the larger assembly span generated by CLC compared to our test Velvet and ABySS runs. The program also discards read tracking information to reduce the amount of time and memory needed, and as a result there is no direct way to verify the assembly. It is a closed-source application and has only one user-definable assembly parameter (minimum output contig length). Even though the CLC assembly for our data was comparable to the others in tests for novel sequence and alignments to reference sequences, we cannot currently recommend it for a transcriptome assembly project because (i) alternate transcripts cannot be assembled, (ii) there is no way of knowing where each read was placed, and (iii) assembly parameters cannot be explored to get a better assembly. However, because of its speed, it is a very useful tool to quickly estimate the quality of a read set.

Roche 454’s own software Newbler has been the workhorse of 454 pyrosequencing transcriptome assembly projects [1-14]. The greatest apparent advantage of Newbler is that it has been designed to deal with transcriptome assemblies (using the –cdna option), where it creates isotigs, sets of contigs that are inferred to represent alternate transcripts. Newbler splits initial contigs wherever the read overlap graph branches, and then stitches these back together into isotigs using reads that connect contigs. However, Newbler (Version 2.3) creates assemblies that are up to 40% smaller than other assemblers and this loss of assembly span explains its poor performance in terms of novel sequence and alignments to reference sequences. Newbler (Version 2.5) addresses these issues and provides a new option (-urt) that does not discard contigs with low bridging coverage (see Methods for a description of this feature). This new option will be available in the next public release of Newbler.

Although CAP3 did not perform best in any of our measures, it aligned to reference sequences about as well as did MIRA and CLC. It has many user-definable parameters and works well with paired sequences, so researchers who are familiar with CAP3 will probably want to continue to use it because they will have a better understanding of how to vary parameters to get assemblies that are possibly better than the other programs we have studied.

**References**

1. **Sequence assembly with MIRA3**. 2010. [http://mira-assembler.sourceforge.net/docs/DefinitiveGuideToMIRA.html]

2. **CLC bio: CLC Assembly Cell User Manual**. [http://www.clcbio.com/index.php?id=1393]
